# Exploring the Relationship of Bone Turnover Markers and Bone Mineral Density in Community-Dwelling Postmenopausal Women

**DOI:** 10.1155/2021/6690095

**Published:** 2021-04-21

**Authors:** Xu Wei, Yili Zhang, Xinghua Xiang, Menghua Sun, Kai Sun, Tao Han, Baoyu Qi, Yanming Xie, Ranxing Zhang, Liguo Zhu

**Affiliations:** ^1^Wangjing Hospital, China Academy of Chinese Medical Sciences, Beijing, China; ^2^Institute of Orthopaedics of Beijing Integrative Medicine, Beijing, China; ^3^Beijing University of Chinese Medicine, Beijing, China; ^4^Hunan University of Science and Technology, Xiangtan, China; ^5^Institute of Basic Research in Clinical Medicine, China Academy of Chinese Medical Sciences, Beijing, China; ^6^Department of Clinical Laboratory, Eye Hospital, China Academy of Chinese Medical Sciences, Beijing, China

## Abstract

**Aims:**

To explore the relationships of procollagen type 1 N-terminal propeptide (P1NP) and *β* cross-linked C-telopeptide of type 1 collagen (*β*-CTX) with bone mineral density (BMD) in postmenopausal women.

**Methods:**

All postmenopausal women were selected from a community-based case-control study. The anteroposterior L1-L4 and left proximal femur BMD were measured. P1NP and *β*-CTX were also collected and tested. The main correlation analysis was applied to explore the relationships of BMD, P1NP, and *β*-CTX.

**Results:**

The total 1055 postmenopausal women were enrolled. The BMD at all sites kept a decrease continually with age (*P* < 0.01). In addition, the level of *β*-CTX increased significantly from 45 to 50 years old and remained at a high level in the later stage, while the level of P1NP changed little or even decreased with age. Logistic regression model showed that *β*-CTX has better ability to predict BMD than P1NP, as demonstrated by an area under the curve (AUC) of 0.63.

**Conclusion:**

P1NP and *β*-CTX are important markers to monitor bone metabolism. This trial is registered with ChiCTR-SOC-17013090. The date of registration is Oct. 23, 2017.

## 1. Introduction

The most frequently used tool to diagnose osteoporosis (OP), the efficacy evaluation, and predict fracture risk is bone mineral density (BMD) in different sites according to the criteria of the World Health Organization [1]. However, the changes in BMD values are very small within six months and very difficult to detect acute changes in bone turnover [2, 3]. On the contrary, bone turnover markers (BTMs) could identify changes in bone remodeling within a relatively short-time interval before changes in BMD can be detected [4, 5]. The values of BTMs are often used to assess the treatment options and efficiency of antiresorptive anabolic therapies or combination therapies [6]. In postmenopausal osteoporosis (PMOP), levels of bone resorption markers above the upper limit of the premenopausal range are associated with an increased risk of fracture [7]. Moreover, skeletal turnover is easily and noninvasively evaluated by the measurement of serum or urinary biochemical BTMs [8].

In all serum bone formation and resorption indices, two specific markers are the most recognized in the OP research: procollagen type 1 N-terminal propeptide (P1NP) and *β* cross-linked C-telopeptide of type 1 collagen (*β*-CTX) [9]. P1NP is a serum biomarker of bone formation, while *β*-CTX is a biomarker of bone resorption [10]. The International Osteoporosis Foundation and the International Federation of Clinical Chemistry and Laboratory Medicine recommended that P1NP and *β*-CTX were used as the predictor of fracture risk and monitoring of OP treatment as early as 2011 [11, 12]. Based on the background, the research on P1NP and *β*-CTX in the diagnosis and treatment of OP has attracted more attention than ever. Biochemical bone turnover markers have been already recommended in the national OP clinical practice guideline or consensus documents [13, 14].

Several studies were conducted to explore BTMs in Chinese populations, especially the P1NP and *β*-CTX levels. A community-based population study was designed to evaluate reference ranges of P1NP and *β*-CTX in healthy Beijing postmenopausal women [15]. In 2013, the levels of P1NP and *β*-CTX were measured in a healthy Shanghai population covering premenopausal and postmenopausal women [16]. Another Chinese study analyzed 1436 healthy volunteers in 5 Chinese cities, and the relation of BMD and BTMs was evaluated in a large healthy Chinese population [17]. A high incidence of OP and osteoporotic fractures was demonstrated in the community-dwelling middle-aged and aged people [18, 19]. Nevertheless, there is little information on the levels of P1NP and *β*-CTX and their relationship with BMD in community-dwelling postmenopausal women in Beijing, China. Accordingly, the case-control study exploring the relation between biochemical indicators and bone mass state in postmenopausal women is required.

## 2. Methods

### 2.1. Study Design

This is a case-control study as a part of BEYOND study (BEijing communitY-based Osteoporosis and osteoporotic fracture screening: a cross-sectioNal and prospective stuDy), starting in November, 2017 [20]. The study protocol was registered in the Chinese Clinical Trial Registry center (registration number: ChiCTR-SOC-17013090). A total of 1642 community residents who lived in Chaoyang and Dongcheng Districts of Beijing City were contacted via community health centers and recruited by clustered sampling in the baseline survey. It was conducted in November 2017 to July 2018 from local communities. In the present study, all postmenopausal women were selected from the surveyed population.

### 2.2. Ethical Statement

The authors stated that this study was approved by the medical ethics committee, Wangjing Hospital, China Academy of Chinese Medical Sciences (approval number: WJEC-KT-2017-020-P001) and followed the principles outlined in the Declaration of Helsinki for all human. In addition, for the investigations involving human subjects, a written informed consent has been obtained from the participants involved.

### 2.3. Study Participants

**Figure 1:**
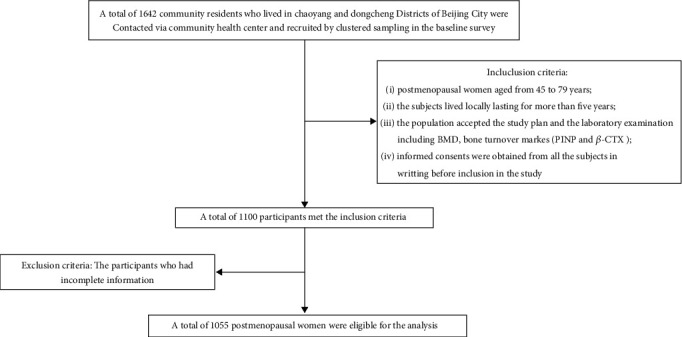
Flowchart of the subject selection process. BMD: bone mineral density; P1NP: procollagen type 1 N-terminal propeptide; *β*-CTX: *β* cross-linked C-telopeptide of type 1 collagen.

Figure 1 depicts a flowchart for participant selection in our study. All the subjects underwent careful past medical history inquiry and physical examination. The inclusion criteria in our study were as follows: (1) postmenopausal women aged from 45 to 79 years; (2) the subjects lived locally lasting for more than five years; (3) the population accepted the study plan and the laboratory examination including BMD and bone turnover markers (P1NP and *β*-CTX); and (4) informed consents were obtained from all the subjects, in writing, before inclusion in the study. The participants in the total population who had incomplete information were excluded from the study. Eventually, 1055 postmenopausal women were eligible and enrolled in the present analysis.

### 2.4. Interviews

All the participants were interviewed via a standardized questionnaire to collect information and completed a face-to-face paper version of questionnaire. The content mainly contained age, weight, height, and time since menopause, history of previous illness, lifestyles, and so on. The comorbidity including cerebral infarction, coronary heart disease, dyslipidemia, hypertension, and diabetes mellitus was mainly reported by the subjects based on the currently prescribed medications. In addition, the lifestyles consisting of current smoking, habitual drinking, regular exercise, milk intake, and coffee intake were also investigated.

### 2.5. Bone Mineral Density Measurement

Dual-energy X-ray absorptiometry device (Hologic, WI, USA) was used to assess the value of BMD (g/cm^2^). The anteroposterior L1-L4 and left proximal femur including the femoral neck and the total hip BMD were detected, and the *T* and *Z* values of each site were also recorded. According to the WHO diagnosis criteria, *T* − score > −1 was defined as normal bone mass, *T* − score ≤ −1 and >-2.5 was defined as osteopenia; whereas, *T* − score ≤ −2.5 was defined as OP based on bone densitometry [21]. After using the instrument daily, a professional staff was responsible for measuring the accuracy and debugging problems. The DXA scanner was calibrated every day, and the coefficient of variability values of the instrument was set at around 1%.

### 2.6. Bone Turnover Markers Testing

Fasting blood samples of the participants were collected between 8 a.m. and 9 a.m. in the sitting position. And the venipuncture was done in the antecubital region with minimal venostasis for the testing of P1NP and *β*-CTX. The measurements were conducted through automated electrochemiluminescence immunoassay system (Roche, Cobas E601, Germany), conforming to laboratory quality control procedures in the clinical practice guidelines of bone metabolic biomarkers (WS/T 357-2011) issued by National Health Commission of the People's Republic of China [22]. In addition, the serum segregated for detection was stored at -80 centigrade freezer. As a professional third-party testing organization, Guangzhou Kingmed Diagnostics Limited Liability Company was responsible for collecting and testing blood samples.

### 2.7. Statistical Analysis

The continuous variables which satisfied normal distribution were presented as the means ± standard deviations. Data that did not show a normal distribution were expressed as the median [interquartile range (IQR)]. Categorical variables were represented by frequency and percentage (%). The comparison for each examined index between the confirmed OP and non-OP (osteopenia and normal) population used Student's *t*-test or one-way analysis of variance (ANOVA). The best-fitting mathematical model was applied to analyze the relationships between BMD and BTMs. Univariate and multivariate logistic regression analyses were used to evaluate the predictive value of bone resorption (*β*-CTX) and formation (P1NP) for BMD. Furthermore, the ability of *β*-CTX and P1NP to identify BMD was assessed through receiver operating characteristic (ROC) curve analysis. All statistical results were analyzed by SPSS 23.0 software (SPSS Inc., Chicago, IL, USA), and *P* values ≤ 0.05 were considered as statistically significant.

## 3. Results

### 3.1. Subject Characteristics

Table 1 presents the characteristics of participants in the study. The basic anthropometry, BMD, BTMs, history or comorbidity, and lifestyle factors were described. The study population was composed of four parts: total population, confirmed OP population, osteopenia population, and normal population. In our study, 432 subjects were diagnosed OP, and the confirmed OP population became the older age group and had a lower body mineral index and longer menopausal duration (*P* < 0.01). The level of P1NP and *β*-CTX in the nonosteoporosis group (osteopenia group and normal group) was significantly lower than that of the confirmed OP group (*P* < 0.01). In addition, the confirmed OP group also occupied a higher proportion in the history of cerebral infarction (*P* = 0.01).

### 3.2. BMD, P1NP, and *β*-CTX Values in Different Age Groups

**Figure 2:**
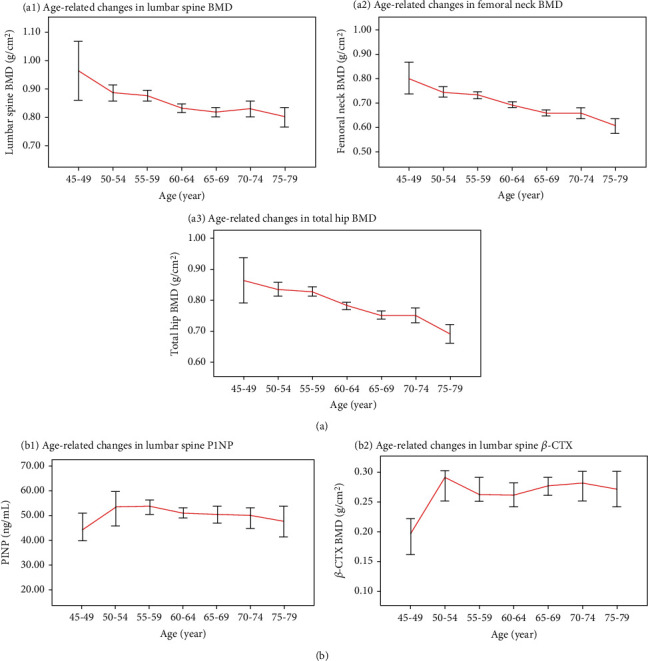
Age-related changes in BMD, P1NP, and *β*-CTX. BMD: bone mineral density; P1NP: procollagen type 1 N-terminal propeptide; *β*-CTX: *β* cross-linked C-telopeptide of type 1 collagen.

Table 2 shows the BMD, P1NP, and *β*-CTX values at lumbar spine, femoral neck, and the total hip in different age groups. The BMD at all sites kept a decrease continually with age (*P* < 0.01). In addition, the level of *β*-CTX increased significantly from 45 to 50 years old and remained at a high level in the later stage, while the level of P1NP changed little or even decreased with age (Figure 2). The results from the aged 45 to 79 groups confirmed that a relative BMD decrease of 17%, 25%, and 21% was found at lumbar spine, femoral neck, and the total hip, respectively. The results from the aged 45 to 79 groups confirmed that a relative BTM increase of 8% and 35% was found at P1NP and *β*-CTX, respectively.

### 3.3. Correlations between P1NP, *β*-CTX, and BMD

**Figure 3:**
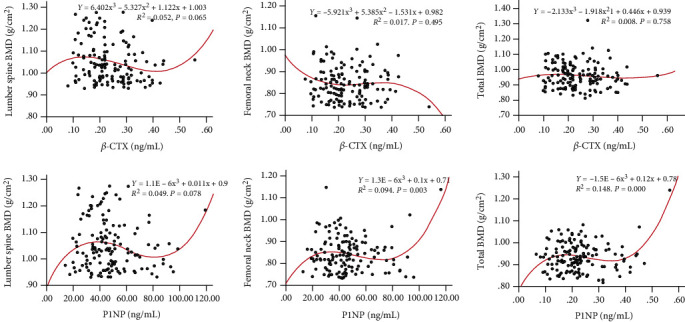
Correlations of bone turnover markers and BMD at all sites in normal group under the cubic model. BMD: bone mineral density; P1NP: procollagen type 1 N-terminal propeptide; *β*-CTX: *β* cross-linked C-telopeptide of type 1 collagen.

**Figure 4:**
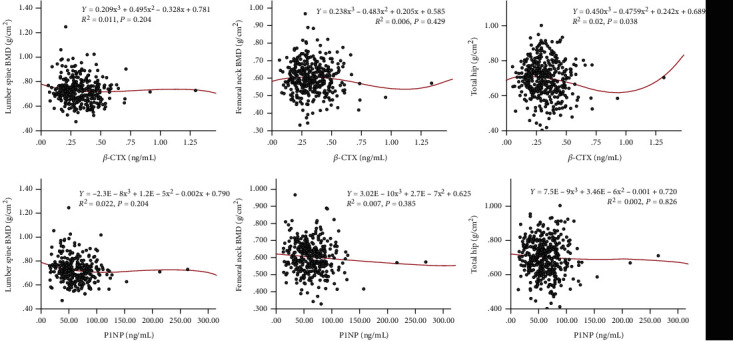
Correlations of bone turnover markers and BMD at all sites in confirmed osteoporosis group under the cubic model. BMD: bone mineral density; P1NP: procollagen type 1 N-terminal propeptide; *β*-CTX: *β* cross-linked C-telopeptide of type 1 collagen.

Table 3 depicts the correlations between the BMD and BTMs, which found that *β*-CTX was negatively correlated with lumbar spine BMD in the normal group and negatively correlated with total hip BMD in the confirmed OP group (*P* < 0.05). Moreover, Spearman analysis also showed that P1NP had a significantly negative correlation with lumbar spine BMD (*P* < 0.01) in OP population. In addition, Figures 3 and 4 depict the correlations of bone turnover markers and BMD at all sites in different population under the cubic model.

### 3.4. Predictive Value of BTMs (P1NP and *β*-CTX) for BMD

**Figure 5:**
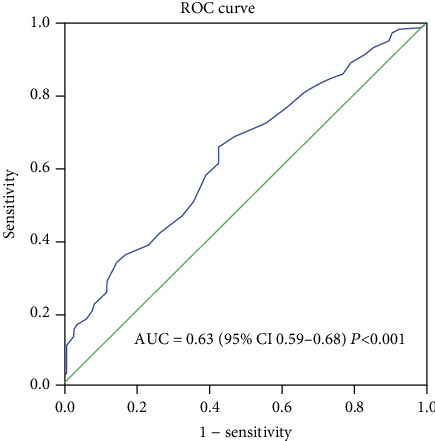
Receiver operating characteristic curves displaying the predictive performance of *β*-CTX in OP patients. ROC: receiver operating characteristic; AUC: area under the ROC curve; CI: confidence interval.

Logistic regression was used to investigate the value of the biomarkers of bone resorption and formation in the prediction of BMD (Table 4). In the univariate analysis, P1NP and *β*-CTX were significant predictors of BMD. Subsequently, these two indicators value with *P* < 0.05 were included in the multivariate logistic regression (forward) analysis, which revealed that only *β*-CTX was a significant independent predictor of BMD (odds ratio = 39.56, 95% CI: 3.7-422.94; *P* = 0.002). ROC curve analysis was then performed to evaluate the independent predictors of BMD (Figure 5). *β*-CTX was the best predictor for BMD, as demonstrated by an area under the curve (AUC) of 0.63.

## 4. Discussion

The association between BTMs and BMD is controversial, due primarily to discrepancy in findings. Moreover, most previous studies were conducted in Caucasian populations [23], with very few studies being done on Asian populations [24]. Thus, the novelty of this study was that we wanted to evaluate the association between BTMs (P1NP and *β*-CTX) and BMD in a sample of Chinese women with wide-age groups and explored the contribution of these markers to the variation in BMD.

This case-control study showed that the level of BMD in postmenopausal women was lower than that in premenopausal women. From the viewpoint of different age group, the BMD value at all sites kept a decrease continually with age (Table 2). Previous study has been indicated that the organic components of bones are mainly composed of type I collagen (about 90%), bone-binding proteins (about 10%), and other trace proteins [25]. Bone development stops after puberty, but cellular activity (bone remodeling) continues to maintain a dynamic balance between bone formation and bone resorption. However, menopause and certain pathological processes may upset this balance and lead to OP. Our study showed that P1NP and *β*-CTX have a relative increasing trend in the early postmenopausal period (Figure 2), which concurred with the results of Vasikaran et al. [26] and Lou et al. [27].

In the organic components of bone matrix, type I collagen is synthesized by osteoblasts, and its N-terminus is P1NP. The increased activity of osteocytes could drive the synthesis of procollagen and increase the blood concentration of P1NP. Hence, the concentration of P1NP in blood can be used as a marker to reflect the ability of osteoblasts to synthesize collagen, which is also the basis for evaluating osteoblast activity and bone formation [28, 29]. *β*-CTX, the degradation product of C-terminal peptide of type I collagen, is one of the most valuable markers to evaluate osteoclast activity and bone resorption [30, 31], and its increase could reflect the degree of bone resorption. In our study, we found that the level of *β*-CTX increased significantly from 45 to 50 years old and remained at a high level in the later stage, while the level of P1NP changed little or even decreased with age, which indicated that the degree of bone resorption was greater than bone formation. Similar study confirmed that it was not only consistent with the development trend of BMD but also may explain the reason for the decrease of bone mass [32].

Estrogen research has provided landmark research on understanding the relationship between osteoporosis and BTMs [33]. Declining circulating estradiol levels, particularly during the menopausal transition, gives rise to increased bone turnover, causing an imbalance between bone resorption and formation [34, 35]. With the extension of time since menopause, the positive correlation of estrogen with BMD and BTMs was found in our study. Moreover, during the aged 45 to 59, the fluctuation of BTMs was more obvious, reflecting that estrogen concentration was one of the most important factors determining BTMs.

Estrogen loss promotes osteoclast formation and bone resorption while inhibiting osteoclast apoptosis through a variety of mechanisms. In the case of withdrawal of estrogen after menopause, the expression of RANKL (a molecule essential for osteoclast formation) in osteoblast lineage including mesenchymal stem cells [36], osteocytes [37], and bone lining cells [38] increased, while OPG production decreased. The increased RANKL could bind to RANK and induce the recruitment of TNF receptor-associated factor 6 (TRAF6), and further activate the downstream NF-*κ*B and MAPK pathways; both of which drive the activation of NFATc1 and stimulate osteoclast formation [39]. Estrogen deficiency also inhibits osteoclast apoptosis by inhibiting Fas/FasL system [40]. In contrast to postmenopausal bone resorption, bone formation decreased relatively. In its physiological state, estrogen could protect osteoblasts from apoptosis and enhance their proliferation, maturation, and mineralization to maintain bone formation through various signaling pathways [41, 42]. However, these osteoprotective effects are counteracted by estrogen deprivation. Furthermore, previous study revealed that ovariectomy-induced estrogen deficiency stimulated the activation of NF-*κ*B in differentiated osteoblasts, thus weakening the function of osteoblasts [43].

It is worth noting that previous studies have confirmed that both types of BTMs (resorption and formation) are more increased in early postmenopausal period due to accelerated bone resorption [14, 44]. BTMs still increase in elderly patients, which is usually explained by other mechanisms (vitamin D deficiency, intestinal malabsorption of calcium, and secondary hyperparathyroidism) [45]. In addition, previous studies have confirmed that BTM is correlated with BMD value in different skeleton sites [46, 47]. However, we did not observe the negative correlation of BTMs and BMD value at all sites (Table 3 and Figures 3 and 4) in the present study. However, given the limitations of sample size and study period, the negative correlation between BMD and BTMs has not been observed in this study, which may need to be confirmed by further follow-up studies.

## 5. Strengths and Novelty

Our study has the following novelty and strengths: (1) as far as we know, this study was the first time to analyze the correlation between BTMs and BMD in Beijing area, which may further deepen and expand the existing evidence [16]; (2) the sample size was large enough and the study participants with a wide age-range were recruited from the urban and suburb, thereby avoiding sample error and being representative of the Chinese population; (3) this study utilized current recommendations and gold standards to evaluate BMD along with prevalence of osteopenia and osteoporosis; and (4) we well descripted and screened the participants' characteristics, and have the detailed inclusion and exclusion criteria to ensure a more precise sample population.

## 6. Limitations

This study did, however, have some limitations. The main limitation of this study was the observational study design with group comparisons, which did not enable causal interpretations. Moreover, BTMs were also affected by dietary and circadian rhythms [48], and the limited number of BTMs analyzed showed an incomplete process of bone metabolism. Therefore, we will continue to expand the sample size for follow-up study to explore the deeper relationship between BTMs and BMD in a larger population.

## 7. Conclusion

The low bone mass state was significantly associated with the increased levels of BTMs in the development of osteoporosis. For the community-dwelling postmenopausal women with different age, P1NP and *β*-CTX were important markers to monitor bone metabolism. Considering the findings in this study, more future extensive studies are necessary to clarify potential molecular mechanisms to help develop more effective therapeutic interventions that may slow the progression of postmenopausal osteoporosis.

## Figures and Tables

**Table 1:** Characteristics of subjects in the study.

Variables	All participants (*N* = 1055)	Confirmed OP (*N* = 432)	Osteopenia (*N* = 481)	Normal (*N* = 142)	*P* value for difference	
					Unadjusted^a^	Adjusted^b^
Age (years)	63.14 (6.72)	65.06 (6.62)	62.33 (6.41)	60.06 (6.35)	<0.001	
BMI (kg/m^2^)	25.32 (3.35)	24.41 (3.30)	25.65 (3.20)	26.97 (3.13)	<0.001	
Time since menopause	13.36 (7.71)	15.76 (7.70)	12.51 (7.24)	9.69 (6.73)	<0.001	
Lumbar spine BMD (g/cm^2^)	0.84 (0.14)	0.73 (0.09)	0.88 (0.08)	1.05 (0.09)	<0.001	<0.001
Femoral neck BMD (g/cm^2^)	0.69 (0.12)	0.61 (0.09)	0.71 (0.08)	0.85 (0.08)	<0.001	<0.001
Total hip BMD (g/cm^2^)	0.79 (0.13)	0.70 (0.10)	0.82 (0.08)	0.96 (0.08)	<0.001	<0.001
P1NP (ng/mL)	52.73 (41.02, 68.95)	56.98 (45.41, 74.72)	50.75 (39.70, 66.28)	45.20 (34.64, 55.15)	<0.001	<0.001
*β*-CTX (ng/mL)	0.27 (0.20, 0.35)	0.29 (0.23, 0.39)	0.25 (0.20, 0.33)	0.22 (0.18, 0.30)	<0.001	<0.001
History or comorbidity (%)						
Cerebral infarction	81 (7.68%)	45 (10.42%)	32 (6.70%)	4 (2.80%)	0.01	
Coronary heart disease	126 (11.94%)	57 (13.19%)	56 (11.70%)	13 (9.20%)	0.42	
Dyslipidemia	267 (25.31%)	110 (25.46%)	119 (24.90%)	38 (26.80%)	0.90	
Hypertension	450 (42.65%)	178 (41.20%)	213 (44.60%)	59 (41.5%)	0.58	
Diabetes (type I)	3 (0.30%)	3 (0.70%)	0 (0.00%)	0 (0.00%)	0.12	
Diabetes (type II)	193 (18.40%)	77 (17.90%)	86 (18.00%)	30 (21.10%)	0.66	
Lifestyle factors (%)						
Current smoking	50 (4.74%)	20 (4.63%)	27 (5.60%)	3 (2.10%)	0.45	
Habitual drinking (≥once/week)	55 (5.21%)	18 (4.17%)	29 (6.00%)	8 (5.60%)	0.31	
Regular exercise (≥3 times/week)	40 (3.79%)	17 (3.94%)	17 (3.70%)	6 (4.60%)	0.62	
Milk intake (≥3 times/week)	731 (69.29%)	285 (65.97%)	350 (72.80%)	96 (67.60%)	0.07	
Coffee intake (≥3 times/week)	33 (3.13%)	7 (1.62%)	20 (4.20%)	6 (4.20%)	0.25	

*N*: number of subjects; OP: osteoporosis; BMI: body mass index; BMD: bone mineral density; P1NP: procollagen type 1 N-terminal propeptide; *β*-CTX: *β* cross-linked C-telopeptide of type 1 collagen. Values are presented as the mean (standard deviation) or prevalence (%). ^a^*P* values were obtained by Student's *t*-test, Kruskal-Wallis test, or chi-square test. ^b^*P* values were obtained by analysis of covariance.

**Table 2:** BMD at all sites, P1NP, and *β*-CTX values in different age groups.

Age group (years)	Lumbar spine BMD	Femoral neck BMD	Total hip BMD	P1NP	*β*-CTX
45-49 (*N* = 12)	0.964 (0.164)	0.802 (0.104)	0.880 (0.122)	45.40 (41.40, 51.71)	0.20 (0.16, 0.22)
50-54 (*N* = 102)	0.885 (0.143)	0.747 (0.110)	0.849 (0.122)	55.12 (41.85, 76.14)	0.29 (0.20, 0.36)
55-59 (*N* = 205)	0.875 (0.132)	0.733 (0.107)	0.842 (0.115)	55.53 (44.83, 70.60)	0.26 (0.21, 0.34)
60-64 (*N* = 290)	0.831 (0.132)	0.692 (0.105)	0.793 (0.111)	52.68 (39.72, 67.97)	0.26 (0.20, 0.34)
65-69 (*N* = 251)	0.817 (0.134)	0.659 (0.101)	0.761 (0.113)	52.11 (40.87, 69.16)	0.28 (0.20, 0.36)
70-74 (*N* = 135)	0.828 (0.163)	0.658 (0.135)	0.760 (0.148)	51.56 (38.61, 64.37)	0.28 (0.22, 0.36)
75-79 (*N* = 60)	0.799 (0.135)	0.605 (0.114)	0.696 (0.119)	49.17 (36.07, 64.34)	0.27 (0.21, 0.34)
Statistics	8.05	23.19	21.90	8.58	11.80
*P*	<0.001^a^	<0.001^a^	<0.001^a^	0.20^b^	0.07^b^

*N*: number of subjects; BMD: bone mineral density; P1NP: procollagen type 1 N-terminal propeptide; *β*-CTX: *β* cross-linked C-telopeptide of type 1 collagen. ^a^*P* values were obtained by one-way ANOVA. ^b^*P* values were obtained by the Kruskal-Wallis test.

**Table 3:** Correlations between BMD, P1NP, and CTX.

	Variables	Lumbar spine BMD		Femoral neck BMD		Total hip BMD	
		*r*	*P*	*r*	*P*	*r*	*P*
Normal (*N* = 142)	P1NP	-0.09	0.31	-0.02	0.80	0.03	0.69
	*β*-CTX	-0.20	0.02	-0.04	0.60	-0.11	0.20
Confirmed OP (*N* = 432)	P1NP	-0.15	0.002	-0.07	0.14	-0.04	0.40
	*β*-CTX	-0.08	0.09	-0.05	0.27	-0.14	0.004

BMD: bone mineral density; P1NP: procollagen type 1 N-terminal propeptide; *β*-CTX: *β* cross-linked C-telopeptide of type 1 collagen; *r*: correlation coefficient. *P* values were obtained by Spearman correlation analysis.

**Table 4:** Univariate and multivariate logistic regression analyses of P1NP and *β*-CTX for predicting BMD.

BTMs	Univariate logistic			Multivariate logistic (enter)		
	OR	95% CI	*P* value	OR	95% CI	*P* value
P1NP	1.02	(1.01, 1.03)	<0.001			
*β*-CTX	158.24	(22.99, 1089.33)	<0.001	39.56	(3.70, 422.94)	0.002

Data was presented by *P* value, OR, and 95% CI. The value of BTMs to predict BMD was tested using a univariate and multivariate logistic regression model. *P* value < 0.05 was considered statistically significant. BMD: bone mineral density; P1NP: procollagen type 1 N-terminal propeptide; *β*-CTX: *β* cross-linked C-telopeptide of type 1 collagen; CI: confidence interval.

## Data Availability

All data used to support the findings of this study are included within the article.
